# Enhancing the Responsiveness of Thermoelectric Gas Sensors with Boron-Doped and Thermally Annealed SiGe Thin Films via Low-Pressure Chemical Vapor Deposition

**DOI:** 10.3390/s24103058

**Published:** 2024-05-11

**Authors:** Woosuck Shin, Maiko Nishibori, Toshio Itoh, Noriya Izu, Ichiro Matsubara

**Affiliations:** National Institute of Advanced Industrial Science and Technology (AIST) Sakurazaka, Moriyama-ku, Nagoya 463-8560, Japan

**Keywords:** SiGe thin film, Seebeck coefficient, thermal conductivity, thermoelectric gas sensor

## Abstract

Thermoelectric gas sensor (THGS) devices with catalysts and Si_0.8_Ge_0.2_ thin films of different boron doping levels of 10^18^, 10^19^, and 10^20^ cm^−3^ were fabricated, and their transport properties are investigated. SiGe films were deposited on Si_3_N_4_/SiO_2_ multilayers on Si substrates using low-pressure chemical vapor deposition (LPCVD) and thermally annealed at 1050 °C. The Seebeck coefficients of the SiGe films were increased after thermal annealing, ranging from 191 to 275 μV/K at temperatures of 74 to 468 °C in air, and reaching the highest power factor of 6.78 × 10^−4^ W/mK^2^ at 468 °C. The thermal conductivity of the SiGe films varied from 2.4 to 3.0 W/mK at 25 °C. The THGS detection performance was tested for the H_2_ gas in air from 0.01 to 1.0%, and compared to the thermoelectric properties of the SiGe films. The high-temperature annealing treatment process was successful in enhancing the thermoelectric performance of both the SiGe films and sensor devices, achieving the best THGS performance with the sensor device fabricated from the annealed SiGe film with 10^18^ cm^−3^ boron-doped Si_0.8_Ge_0.2_.

## 1. Introduction

Microscale energy harvesting and refrigeration are becoming highly attractive for microelectronic and optoelectronic applications, for examples, stabilizing optical device temperatures, and improving the reliability and reducing the noise of these devices. To achieve their high performance, thick- or thin-film thermoelectric (TE) material processing is the most important technology. Currently, many TE materials based on bismuth telluride (BiTe) or lead telluride (PbTe) have been developed for various applications. In the microdevice domain, as well as Bi_2_Te_3_, heavily doped films made of silicon–germanium alloys (SiGe and Si) have also emerged for operations at temperatures above room temperature. These thin-film-type high-temperature materials made of SiGe and Si can be prepared using various film preparation techniques, such as the conventional RF-sputtering method [1,2], the low-pressure and/or plasma-enhanced chemical vapor deposition (LPCVD, PECVD) method [3,4,5], and/or other low-temperature layer-exchange techniques [6,7] or electroplating processes [8]. These SiGe films can be doped with both phosphorus and boron, and their thermoelectric performances by with different film compositions have been investigated previously around room temperature [4].

In the applications of microscale energy harvesting and refrigeration, thick or thin Bi_2_Te_3_ films have been used for TE microdevices [9,10]. However, considering its adaptability, the silicon-based fabrication process is a very attractive robust alternative, and micro-TE devices using SiGe films have also been developed in recent years [11,12]. In micropower applications, Thermoelectric properties of SiGe thin films [12] at high temperatures have not been reported clearly and are not well understood, which is very important for enabling wide-temperature-range applications. Unfortunately, for the high-temperature measurement technique for Seebeck coefficient of TE materials has not developed well [13,14]. Recently, to solve this subject, a bulk high-temperature NIST standard material of SRM 3452 (boron-doped Si_0.8_Ge_0.2_) for measuring the Seebeck coefficient has recently been developed [15]. However, there is no stable thin-film-type material reported that could be used as a standard for high-temperature measurements to prove the reliability of this measurement at high temperatures.

Micro-thermoelectric power generators combining catalytic combustion and thermoelectric energy conversion have been reported with both bulk [16] and film [10,17] thermoelectric materials. Besides power generation, a novel calorimetric gas sensor [18,19] has also been presented using the same method of catalytic combustion and thermoelectric conversion. This calorimetric gas sensor is called a thermoelectric gas sensor (THGS) and takes advantage of micro-machined devices by using the oxidation heat of inflammable gas generated by catalytic combustors. Analyzing their thermal characteristics is essential for designing these devices and for improving their sensing performance.

The aim of this study is to evaluate the high-temperature TE properties, including the electrical conductivity, Seebeck coefficient, and thermal conductivity, of the SiGe thin films integrated in THGSs. The SiGe thin film functions as an energy transducer and builds up a high voltage via its large Seebeck effect, and is a promising material considering its compatibility with LSI processes. In this study, high-temperature measurements of the resistivity and Seebeck coefficients of boron-doped Si_0.8_Ge_0.2_ thin films with different doping levels were carried out, and the optimum boron doping level and the effects of thermal annealing as a post-treatment were investigated. The differences between the gas detection performance of the THGS with these SiGe films and the measured TE properties of the films are discussed.

## 2. Materials and Methods

### 2.1. Preparation of TE Thin Films

The SiGe thin films were deposited on a Si_3_N_4_/SiO_2_ multilayer on a 150 mm diameter Si wafer substrate with a thickness of 0.5 mm, using a commercially available LPCVD method at 700 °C under a deposition pressure of several Torr. Deposition was carried out with using silane, germane, and diborane as sources for the preparation of the B-doped Si_0.8_Ge_0.2_ thin films. The gas flow of the boron source during the CVD was controlled to obtain the final composition of B content in the films around three different doping levels of 10^18^, 10^19^, and 10^20^ cm^−3^. The thickness of the films was 410 nm measured by their cross-sectional observation using scanning electron microscope. The carrier concentrations in the SiGe films were varied by the thermal annealing as a post-treatment, which was necessary to activate the charge carriers after the deposition.

All of the Si_0.8_Ge_0.2_ thin films were annealed at 1050 °C for 5 h, with a 150 cm^3^/min argon gas flow. Before the thermal annealing, the top surfaces of the SiGe films were protected with a 300 nm layer of SiO_2_. This was performed to suppress the growth of an unwanted oxide layer during the annealing. The oxidation of SiGe thin films is well known and has been extensively investigated; it is influenced by various parameters, such as the SiGe composition and humidity. If this oxidation occurs, then the composition of the SiGe changes because the Ge atom moves into the SiGe film or GeO_2_ vaporizes out. After the annealing, the surface oxide was removed via a reactive-ion etching process with CHF_3_ and O_2_ gas.

### 2.2. Characterization and Electrical Property Measurements of the Thin Films

The phases and crystallinity of the prepared samples were investigated using X-ray diffraction (XRD) using two standard diffractometers (RINT 2100 V/PC, Rigaku, Tokyo, Japan) with CuK*α* radiation in the 2*θ*-*θ* scan mode. A commercial electrical conductivity and Seebeck coefficient measurement system (RZ20001i, Ozawa Science Co., Nagoya, Japan) with the option of thin-film measurement with four-point pressure-contact electrodes was used for measurements of the conductivity, *σ*, and Seebeck coefficient, *α*, of the thin-film boron-doped SiGe samples from 50 to 480 °C.

**Figure 1:**
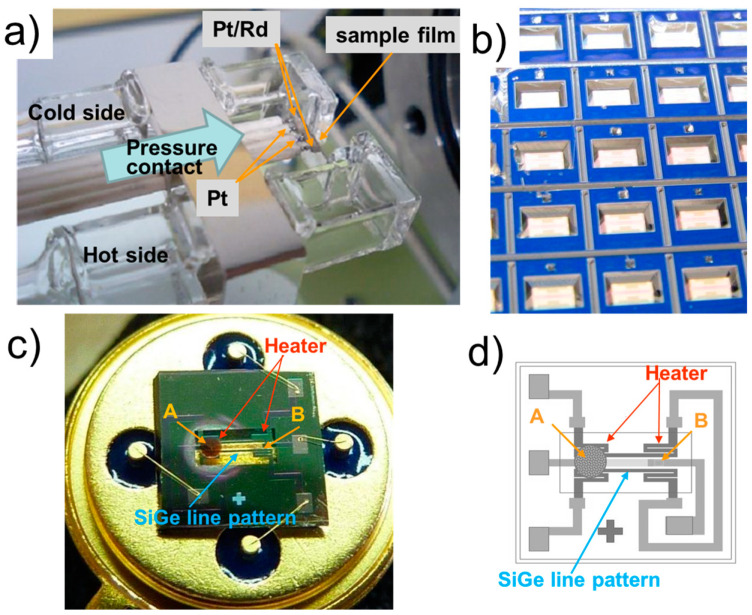
(**a**) Electrodes for the thin-film sample measurement, where the one side of the sample is an air-flow-cooled electrode that induces a temperature difference in the sample. (**b**) A 150 mm Si wafer substrate with a thickness of 0.5 mm after the KOH wet-etching process, showing the back side of the Si wafer and the cavities for the THGS device chips. (**c**) A snapshot of the thermoelectric gas sensor (THGS) with the ceramic catalyst on the left side of the heater, labeled as A. (**d**) Schematic design of the THGS. The unique feature of this sensor is that both the thermoelectric and catalytic parts on a single membrane, i.e., the hot-plates, and the platinum resistor line pattern on the membrane make two meanders. The Pt heater meander triggers the combustion of the hydrogen, which produces heat, and a temperature gradient appears between the hot point A and the cold point B across the SiGe thin-film thermoelectric line pattern.

Figure 1a shows the TE measurement electrode system used in this study with the air-flow-cooled electrode, where one side of the sample is cooled down by air flow inside the double quartz tubes to induce a temperature difference in the sample. This system has been redesigned and we modified its electrode probes to have 4-point pressure-contact electrodes. Every electrode is an R-type thermocouple (TC) of platinum–13% rhodium vs. platinum, which is guided and supported by an alumina tube and pressed onto the thin-film sample. Cooling the cold side of the thin-film sample induces a temperature difference, ΔT, and thermoelectric voltage, ΔV, across the film sample. The measurement error was below 5%, which was verified in a previous study with the same SiGe material and B-doped Si [20].

The Hall carrier density (mobility) of the SiGe thin-film samples was evaluated using Resitest8300 (magnetic field of 0.75T, Toyo Co., Tokyo, Japan) from 25 to 300 °C. The Van der Pauw Hall measurement with the 4-point electrode system was used for this evaluation. The thermal conductivity, κ, of each film sample was measured using the 2ω method (TCN-2ω, Ulvac-riko thermal constant measurement, Yokohama, Japan), which measures the thermal conductivity of each thin film based on the substrate’s depth-direction (cross-plane) thermal conductivity. This method uses both periodical heating using current from the electrode and thermoreflectance (TR) measurements [21,22]. A correction of the Si_3_N_4_/SiO_2_ multilayer on the Si substrate structure was also carried out, as shown in Figures S1 and S2 in the Supplementary Materials. The details of these measurements are also described in the Supplementary Materials.

### 2.3. Micro-THGS Fabrication and Method for Testing Gas Detection

Figure 1b shows the back side of the wafer after the KOH wet-etching process, and the cavities of the sensor devices. As the multilayer Si_3_N_4_/SiO_2_ dielectric membrane is transparent, the SiGe and Pt line patterns on the front side are shown on the bottom of the cavities. Figure 1c,d show the THGS microdevice with the ceramic catalyst deposited on one side of the SiGe line pattern: width × length = 0.18 × 1.2 mm^2^. The thin micro-heater meander films are multilayer sputter depositions of a 10 nm thick Ta adhesion layer, a 200 nm thick Pt layer, and a 10 nm thick Pt-W layer, and were patterned using a lift-off process. The reverse side of the Si substrate was etched out using an aqueous KOH solution to prepare the membrane structure. Details on the processing and patterning of the SiGe thin films have been reported previously [2]. The area of the membrane made through KOH etching was approximately 1 × 2 mm^2^, whereas the heated catalyst area was about 0.65 mm in diameter. The membrane area of the THGS is composed of two ends of thermoelectric SiGe, forming a symmetrical pattern. However, one end of the SiGe pattern is deposited by a Pt/alumina combustion catalyst, which induces an asymmetrical temperature across the membrane, like that shown in Figure 1c,d. The membrane is divided by two points, named A and B, considering this asymmetrical heater meander structure with or without catalyst deposition.

The catalyst on one side of the micro-heater of the Pt meander was heated up to 100 °C during the gas sensor operation. The THGS was mounted on a TO5 package, and the resistance of the THGS was 2~3 kΩ. The gas response performance of the THGS was investigated using a gas-flow-type test chamber. Hydrogen gas diluted in air from 0.01 to 1.0% was flowed into the test chamber that the device was set into. Two flows of the hydrogen gas and air were alternately switched through a measurement chamber with a flow rate of 200 cm^3^/min, and the gas response of THGS was investigated. A temperature difference between the two points of A and B on the THGS device was developed using the heat of gas combustion at A, and TE voltage was generated between the hot and the cold points, A and B, and measured as ΔV using the THGS. An infrared camera (LAIRD-270A, Nikon, Tokyo, Japan) monitored the surface temperature of the catalyst, the hot point A, and the cold point B of the SiGe line on the device, as shown in Figure 1c,d, to evaluate the Δ*T* induced by the gas combustion heat from the catalyst.

## 3. Results and Discussions

### 3.1. Analysis of Crystalline SiGe Thin Films Using XRD

**Figure 2:**
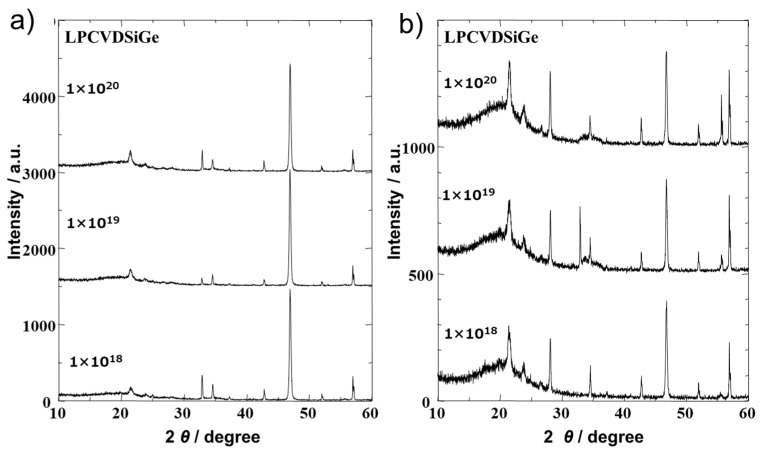
XRD patterns of the boron-doped Si_0.8_Ge_0.2_ thin films (**a**) as deposited via LPCVD and (**b**) after annealing at 1050 °C for 5 h with a 150 cm^3^/min Ar gas flow. The figures shows the nominal boron doping level during the CVD process.

X-ray diffraction (XRD) analysis was used to determine the crystallographic structures of the boron-doped Si_0.8_Ge_0.2_ thin films with different boron doping levels of 10^18^, 10^19^, and 10^20^cm^−3^ deposited on the Si_3_N_4_/SiO_2_/Si substrate, both as deposited and after thermal annealing. Figure 2a shows that the as-deposited SiGe thin films have a diamond crystal structure, and a diffraction peak corresponding to the (220) plane with 2Θ located at 47° was the only prominent peak for all of the samples. This suggests that the deposited SiGe films after LPCVD were oriented and the quality of the films was similar to each other regardless of the B content.

After the thermal annealing, the peaks of other SiGe planes became distinct, as shown in Figure 2b. The diffraction peaks corresponding to the (111) and (311) planes with 2Θ located at 28 and 55°, respectively, became strong and others also appeared. From this result, it can be considered that the high-temperature thermal annealing made the orientation of the SiGe grains in the film random and enhanced the grain growth of SiGe in the film at the same time. We could confirm that the surface of the film changed from a mirror-like surface to a rough surface as this was visible to the naked eye, and this change also supports the results of grain growth of SiGe.

### 3.2. Carrier Concentration of SiGe Films Investigated Using Hall Measurements

**Figure 3:**
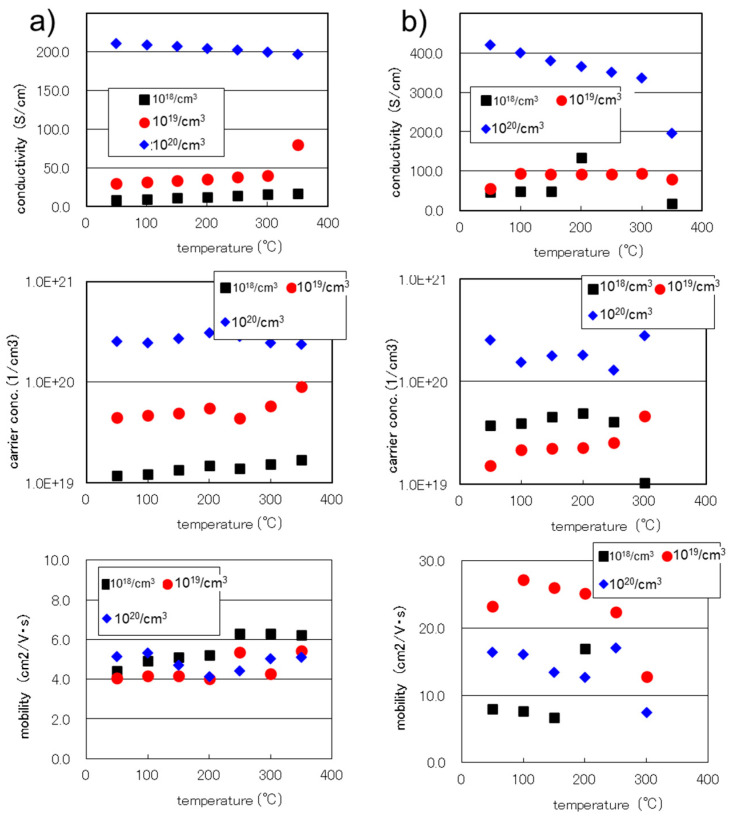
Hall measurement results of the boron-doped Si_0.8_Ge_0.2_ thin films deposited through LPCVD: (**a**) the as-deposited films; (**b**) the films after being annealed at 1050 °C for 5 h with a 150 cm^3^/min argon gas flow. The figure in the box shows the nominal boron doping level during the CVD process.

Unlike the ion implantation process or the well-controlled diffusion process of dopants, it is difficult to control the exact composition of the co-doping during the CVD deposition. In the process of this study, the boron-doping process was relatively controllable and successful, as shown in Figure 3a, as the carrier concentration obtained from the Hall measurements of the Si_0.8_Ge_0.2_ thin films increased linearly.

At 100 °C, the changes in the charge carrier concentrations of the films were 1.22 × 10^19^, 4.67 × 10^19^, and 2.45 × 10^20^ cm^−3^ for the nominal boron compositions of 10^18^, 10^19^, and 10^20^ cm^−3^, respectively, and the mobility values of the three samples were almost at the same level, around 5 cm^2^/Vs. This result that a similar level of carrier mobility for the different doping levels, supports the above discussion. We can understand that the quality of the SiGe crystal was not so much varied by boron doping content in the LPCVD process. We could say that the electrical conductivity, σ, of the as-doped SiGe films increased in the simple carrier-doping process, with the higher boron concentrations resulting in a high conductivity and a high carrier density.

After the thermal annealing, the microstructures of the SiGe films changed as the grains grew, and as a result, the mobility of the thin films increased drastically, as plotted in Figure 3b. The carrier concentrations of the 10^19^ and 10^20^ cm^−3^ films decreased, but that of the 10^18^ cm^−3^ film increased during the annealing. The former could be due to the precipitation of the dopant and the latter could be due to thermal activation, which is a common phenomenon in the post-processing of high-doping-level films. As a result, the conductivities of all the samples increased due to the thermal annealing.

The Hall mobility of the 10^19^ SiGe film after the thermal annealing was around 25 cm^2^/Vs, which is not the best or optimized, but which is as high as that reported elsewhere [23]. Considering the heavy doping levels used in this study, the SiGe films of both a high mobility and high carrier density are a promising achievement of this process. The high mobility of the SiGe film annealed at 1050 °C is roughly five times higher than that of the as-deposited SiGe film or sputter deposited Si_0.8_Ge_0.2_ film [3,24], which were below 5 cm^2^/Vs. Both the high-temperature annealing and the CVD process seem to have enhanced the crystallinity of the films and induce higher carrier mobility. As higher mobility is thought to lead to a better thermoelectric performance, the Seebeck coefficient of a film annealed at a higher temperature is expected to be higher than that of a film annealed at a lower temperature.

### 3.3. Thermoelectric Properties Enhanced through Thermal Annealing

**Figure 4:**
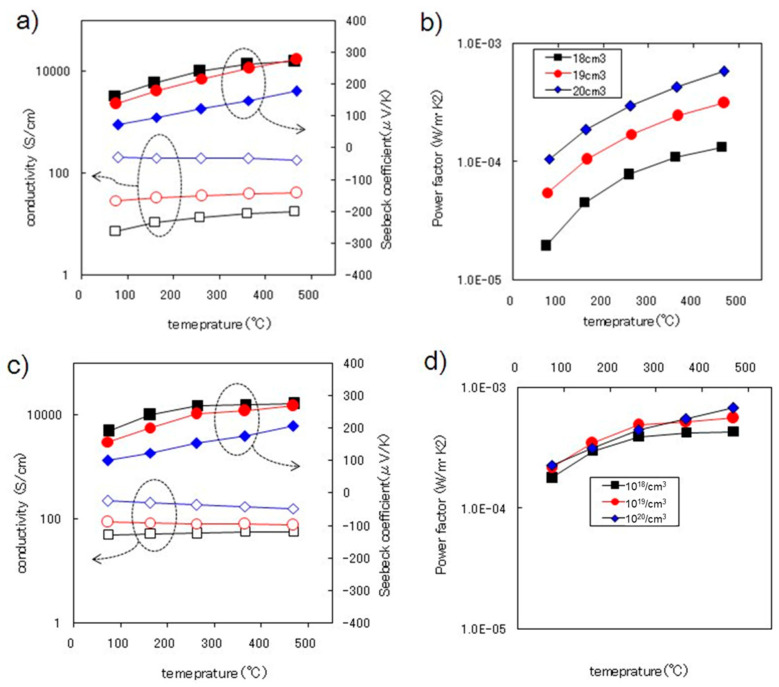
TE measurements of the B-doped SiGe films with different B doping concentrations, 10^18^, 10^19^, and 10^20^ cm^−3^, deposited on the Si_3_N_4_/SiO_2_/Si substrate: (**a**) conductivity and Seebeck coefficient and (**b**) TE *PF* of the as-deposited film, and (**c**,**d**) for the films after thermal annealing.

Figure 4a shows the thermoelectric properties of the as-deposited SiGe films. The Seebeck coefficient, *α*, and the conductivity, *σ*, were measured from 78 to 470 °C in air. The *σ* values deviated a little from the data of the Hall measurements, which seems to be due to both the difference in electrode configurations and the uniformity of the boron dopant in the batch of the thin film. The temperature dependencies of σ measured across a wide temperature range indicate clearly that the films with higher dopant concentrations are metallic, and those of lower dopant concentrations are semiconductor-like. The temperature coefficient of resistance, TCR [25], was large for the films of lower dopant concentrations. However, when increasing the dopant, the TCR of the film decreased and the films became metallic.

The *α* values of all of the SiGe thin films linearly increased with the temperature, ranging from 72 to 278 μV/K, which is the same range as that reported for bulk materials [26,27], and higher than those of the thin film deposited using CVD [28] or sputtering [29]. The *α* of the highly doped SiGe thin film was smaller than the others and was below 200 μV/K even at high temperatures. The power factors, *PF* = *α*^2^*σ*, of the as-deposited films are plotted with respect to the temperature in Figure 4b. The highest *PF* value was obtained for the highly doped SiGe film, 5.80 × 10^−4^ W/mK^2^, at 466 °C. The *PF* of the as-deposited films increased by two times with respect to the ten-fold change in doping level. During the high-temperature thermal annealing, the *α* of the highly doped film increased, as shown in Figure 4c. The *α* of the SiGe thin films with the other two doping levels increased a little and the *σ* of these films increased by two to four times.

The *PF* of these two films increased drastically due to the annealing, as plotted in Figure 4d, and the highest *PF* value was obtained for the highly doped SiGe film, 6.78 × 10^−4^ W/mK^2^ at 468 °C. No serious trade-off in the performance between *σ* and *α* occurred after the annealing process. It can be reasonably understood that the high-temperature annealing resulted in a highly crystalline structure and a higher carrier mobility, which induced larger Seebeck coefficients for the samples. The thermoelectric performances of the films at 78 °C are listed in Table 1, including the thermal conductivity, κ, of the SiGe thin films measured at 25 °C.

The details of the calculation of κ measurements are listed in Table S1 in the Supplementary Materials. Considering the limited number of samples processed in this study, the *PF* of the heavily doped LPCVD SiGe films does not seem to be optimized yet, but other annealing parameters possibly increase the TE performance of the film higher.

The *α* of the 10^18^ and 10^19^ cm^−3^ films decreased at high temperatures during the thermal annealing process in this study, but some processes may suppress this unwanted change. From this discussion, the doping level of 10^19^ cm^−3^ is considered an appropriate doping level and further process modification seems meaningful. The *κ* values of the SiGe thin films were around 2.5 W/mK and were much lower than the reported values for bulk boron-doped SiGe [26] or thin-film SiGe [15].

The *κ* value of the Si_3_N_4_/SiO_2_ multilayer, with thicknesses of 250 and 80 nm, respectively, was checked using same method to be 1.44 W/mK, which is a reasonable level. Commonly, the *κ* increases as a material is prepared into nanostructure, but increases via thermal annealing followed by grain growth. Low values of *κ* were understandably found for the as-deposited SiGe thin films, but there was no noticeable increase caused by the thermal annealing.

### 3.4. THGS Performance: Combustion and Thermal Transport

**Figure 5:**
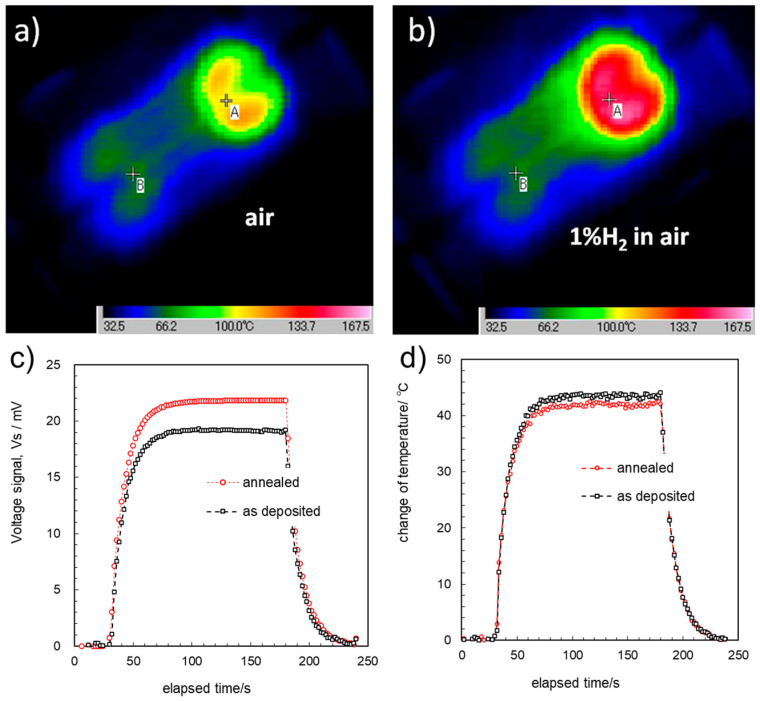
Operando measurements of the gas-sensing performance of THGSs of 10^18^ cm^−3^ boron-doped Si_0.8_Ge_0.2_ thin films: (**a**) temperature distribution of the device surface in air flow; (**b**) in hydrogen 1% air gas flow; (**c**) transient of voltage signal, Vs; (**d**) change in temperature between two points of A and B on the device surface for the devices fabricated with the as-deposited SiGe film and annealed SiGe film.

When the hydrogen–air mixture flows over the THGS device, oxidation of the hydrogen occurs, heating up the catalyst, and a temperature difference between the two points of A and B on the THGS is developed. The temperature distributions of the THGSs were observed with a high-resolution thermal imaging system using the operando method, as shown in Figure 5, which monitored the temperature changes for the device with 10^18^ cm^−3^ boron-doped Si_0.8_Ge_0.2_ films caused by the combustion heat of 1% hydrogen in air. The two heater areas were at almost the same temperature before the hydrogen gas was introduced, as shown in Figure 5a.

When the hydrogen gas was introduced and combustion heat was generated, the temperature of the catalyst of the point A, increased and the heat spread out from the catalyst on the membrane to the Si substrate rim, as shown in Figure 5b. One can observe the variation in temperature along the Pt heater meander, and that the cold side, point B, was heated due to the heat transfer through the SiGe line.

Figure 5c, d show plots of the transient variation of Vs. and ΔT between the two points of A and B on the device. From these IR image data, the temperature changes of the THGS were monitored, as plotted in Figure 5d, where the voltage from the thermoelectric pattern was simultaneously acquired in addition to the thermal images, as plotted in Figure 5c. Comparing these data with the actual device structure, the device pattern can be recognized based on the temperature difference within several degrees. This uncertainty is due to the different IR emission characteristics of different materials or edges of the patterns. When the hydrogen gas flow was introduced, the Vs and ΔT of THGS increased and then saturated at around 30 s, which depended on the flow rate, creating thermal balance. The saturated ΔT of the two devices of as-deposited films and the annealed sample were found to be the same at 43 °C.

### 3.5. Discussion on the Relationship between the Thin Films and the Sensor’s Performance

The ΔT of the THGS was caused by the heat of the catalyst, *Q_c_*, which was generated by the inflammable gas combustion at A, and was measured as Vs using the THGS. The relationship between *Q_c_* and the thermoelectric voltage Δ*V_gas_* can be expressed using the thermal resistances of conduction and convection to the ambient temperatures of sections A and B, *R_A_* and *R_B_*, respectively, by following Equation (1).

(1) $$V_{s}=\Delta V_{gas}=46.1\times {\alpha \cdot R_{A}\cdot Q}_{c}[W/V]$$

Lower thermal conductivity of the SiGe line pattern results in higher *R_A_*, and, as a result, higher voltage signal, Vs. According to the heat capacity for section A, *C_A_*, and the thermal time constant of section A, τ*_A_*= *R_A_* × *C_A_*, [30], the *R_A_* of THGS was estimated to be 230.4 K/mW. The thermal conduction of the device seemed to be similar for these films, but the Vs of the two THGSs with as-deposited and annealed films were 19 mV and 22 mV, respectively. This difference originated from the different doping levels, which is corresponds well to the measured thermoelectric properties. For the THGS devices based on the SiGe films with different boron doping levels and process conditions, the changes in the temperature difference between A and B, ΔT, and Vs were investigated and are listed in Table 2.

**Figure 6:**
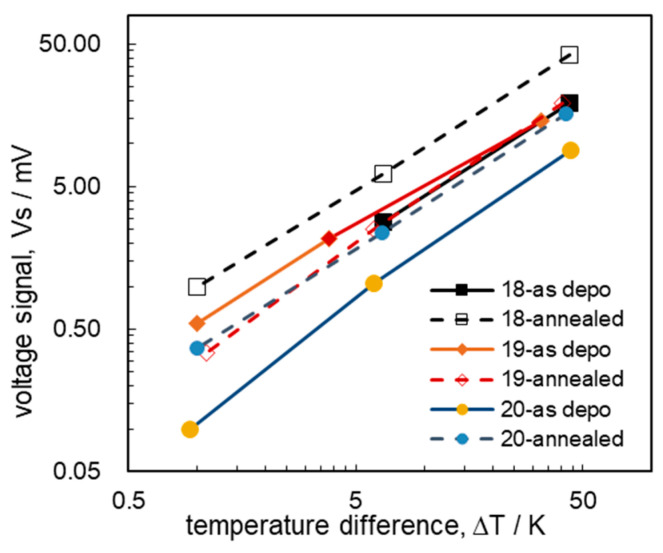
The voltage signals, Vs, of the various THGSs, plotted with respect to the change in the temperature difference between points A and B, ΔT, for different boron doping levels and process conditions.

The ΔT was reduced with the H_2_ gas concentration from 1% to 0.1 and 0.01%. As ΔT changed, Vs varied linearly, showing the typical Seebeck effect. This relationship was established well for all six THGSs, and their voltage signals are plotted in Figure 6. In Table 2, the ΔT values for all six devices are similar to each other, but the different Vs values correspond well to the variation in α for the different SiGe films. However, the apparent α values of the THGS devices from the Vs and ΔT plots in Figure 6 are around 0.3~0.4 mV/K, 150% of the measured values for the SiGe thin films.

This is due to the temperature difference across the thin-film catalyst surface and SiGe film, and the apparent α of THGS is higher than the measured α of the SiGe films. From this plot, we could conclude that the thermal conductivities of the SiGe films are similar to each other and the Seebeck coefficient predominantly effected the Vs of the THGS.

As the process of catalyst combustion for all THGSs in this study was the same process, the effect of the catalyst is considered to be very small. This effect has been studied quantitatively before. The specific heat capacity of the catalyst, estimated according to the catalyst material of Pt/Al_2_O_3_, and its size have been studied in detail via three-dimensional modeling using the finite-element method [31], assuming parameters such as the thermal conductivity of the Si_3_N_4_/SiO_2_/Si membrane to be 10 W/mK. Clear extraction of the thermal conductivity of the multilayer structure was difficult, but this value of the thermal conductivity is roughly close to that of the SiGe film of 3 W/mK measured in this study. As the thermal conductivity of the thin-film SiGe of 3 W/mK is lower than that of the membrane or bulk of SiGe, the effect of the area or thickness of the membrane or the relative design of SiGe line of 0.1 mm becomes more important than the material properties of the SiGe line. A too-thin membrane will reduce the yield of the device during the KOH wet-etching process and create a risk of the device cracking due to thermal shock.

Measuring the thermal properties of the film device is difficult and periodic heating is applied to remove the noise effect [32] and/or free-standing structures [33]. The in-plane thermal conductivity reported by other groups using free-standing bar membranes for the heterolayer-structure LPCVD of SiGe/SiO_2_/Si was 40 W/mK for a boron doping level of 10^19^/cm^3^. The best THGS performance in this study was achieved using the device fabricated with 10^18^ cm^−3^ boron-doped Si_0.8_Ge_0.2_. This is clearly understood, as previous studies [2,4] on the performance of SiGe thin films reported that a higher Seebeck coefficient of the film is more advantageous for the Vs. The THGS with the annealed 10^18^ cm^−3^ boron-doped SiGe showed the highest Seebeck coefficient and a relatively high conductivity. It is also notable that besides the high Seebeck coefficient, the thermal resistance and high conductivity are also important. The conductivity of SiGe could reduce the unwanted noise of the THGS device and be important for accurately detecting low-concentration gases, as less noise means greater sensitivity with signal/noise as the figure of merit. The Johson noise [33] of the THGS at the sampling frequency of 1 Hz can be estimated to be 1 μV at 300 K when the device resistance is 3 kΩ. Considering the Vs of the THGSs with the thermally annealed SiGe films plotted in Figure 6, this Johson noise level was lower than the Vs level for the detection of ppm levels. From this result, our high-temperature annealing treatment process has been successful, especially for the SiGe with the lower doping level of 10^18^ cm^−3^.

## 4. Conclusions

High-temperature measurements of the conductivity, Seebeck coefficient, and thermal conductivity of boron-doped Si_0.8_Ge_0.2_ thin films with different doping levels of 10^18^, 10^19^, and 10^20^ cm^−3^ deposited via LPCVD and thermal annealing has been carried out. Using these SiGe thin films, thermoelectric gas sensor (THGS) devices were fabricated and their performances were investigated. The Seebeck coefficients of the SiGe films were increased after thermal annealing, ranging from 191 to 275 μV/K at temperatures of 74 to 468 °C in air. The highest power factor value for the SiGe films was 6.78 × 10^−4^ W/mK^2^, and the thermal conductivities of the films varied from 2.4 to 3.0 W/mK.

The boron doping worked as a simple carrier-doping process, increasing the carrier concentration, while the thermal annealing process increased the mobility and the α of the lower-doping-level films. The performances of the THGS devices composed of SiGe thin films with a catalytic combustor mostly depended on the α, as the temperature differences caused by the gas combustion were similar to each other. This result is also supported by the similar κ values of the SiGe films around 3 W/mK at 25 °C, and the thermal resistance of the THGSs being similar. The thermal annealing process was successful in enhancing the thermoelectric performance of both the films and THGS devices, and the best THGS performance was achieved with the sensor device fabricated using the annealed SiGe film with 10^18^ cm^−3^ boron-doped Si_0.8_Ge_0.2_.

## Figures and Tables

**Table 1:** Summary of the thermoelectric performances of 6 different boron-doped SiGe films at 78 °C, including as-deposited and annealed films. The κ values of the films were measured at 25 °C.

B Doping Level		α	σ	PF	*κ*	*ZT*
cm^3^		μV/K	S/cm	10^−4^ W/mK^2^	W/m K	
1 × 10^18^	as-deposited	165	7.21	0.196	2.6 ± 0.4	0.025
	annealed	191	49.0	1.79		0.019 *
1 × 10^19^	as-deposited	137	29.3	0.552	2.4 ± 0.6	0.0075
	annealed	159	87.0	2.20		0.024 *
1 × 10^20^	as-deposited	72.0	205	1.06	2.5 ± 0.4	0.014
	annealed	102	222	2.31	3.0 ± 0.5	0.025

* Estimated according to the κ value of 3.0 W/m K.

**Table 2:** Voltage signal Vs and temperature difference ΔT of the THGS sensors fabricated from 6 different SiGe films of various thermoelectric properties.

B Doping Level		α	1% H_2_		0.1% H_2_		0.01% H_2_	
cm^3^		μV/K	Vs/mV	ΔT/K	Vs/mV	ΔT/K	Vs/mV	ΔT/K
1 × 10^18^	as-deposited	165	19.16	43.6	2.81	6.6	0.01	1
	annealed	191	21.8	42.3	3.17	6.2	0.41	1
1 × 10^19^	as-deposited	137	13.35	32.8	1.39	3.8	--	--
	annealed	159	20.67	40	2.14	3.6	--	--
1 × 10^20^	as-deposited	72.0	9.03	38.1	1.04	6.3	0.10	1
	annealed	102	16.1	42.1	2.39	6.5	0.37	1

## Data Availability

Data will be made available on request.

## Supplementary Materials

The following supporting information can be downloaded at https://www.mdpi.com/article/10.3390/s24103058/s1: Figure S1: Measurement principle of the 2ω method for thin films on a substrate. A metal film is deposited on the thin film for Joule heating and TR measurement. (a) Schematic of Joule heat generated by the sinusoidal electrical current and its dissipation toward the substrate; Figure S2: Modified measurement of the 2ω method for thin films on a substrate with SiGe of different thicknesses: (a) 450 nm and (b) 900 nm; Table S1: Thermal conductivity of 3 different boron doping levels for SiGe films on a Si_3_N_4_/SiO_2_ multilayer on a Si substrate.
