# Supplementary material for: Enhancing the Responsiveness of Thermoelectric Gas Sensors with Boron-Doped and Thermally Annealed SiGe Thin Films via Low-Pressure Chemical Vapor Deposition
Source: Sensors (Basel). 2024 May 11;24(10):3058. doi: 10.3390/s24103058 (PMC11124772; doi:10.3390/s24103058)
Supplement: Supplementary file 1 [file sensors-24-03058-s001.zip › sensors-2955425-supplementary.pdf]

Supplementary information

Enhanced response of thermoelectric gas sensors of LPCVD SiGe thin films by boron doping and thermal annealing

Woosuck Shin*, Maiko Nishibori, Noriya Izu, Toshio Itoh and Ichiro Matsubara

National Institute of Advanced Industrial Science and Technology (AIST)

Sakurazaka, Moriyama-ku, Nagoya 463-8560, JAPAN

*Corresponding author, e-mail; w.shin@aist.go.jp

Thermal Conductivity Measurement of Thermoelectric Thin Films by a 2ω Method

The 2ω method is a technique to measure the, κ, of a film sample based on sinusoidal Joule-heating of a metal film deposited on the sample surface and its thermoreflectance (TR) measurement of surface temperature cooling due to the heat dissipation. The 2ω method uses periodic heating of the metal film and subsequent cooling due to heat dissipation to depth, where the temperature of the metal surface is monitored by TR measurement using a continuous wave optical laser. This method is suitable for measuring a cross-plane thermal value of a film sample on a substrate. It is assumed the heat dissipates across a sample film and reaches the substrate in a three-layer system of metal film/thin film/substrate. In this heat dissipation situation, the TR signal is formulated to be an approximate linear function to $(2\omega)^{0.5}$, which linear relationship brings κ values. For this reason, this method is not applicable in the heat dissipation case as the heat flow does not reach the substrate across the high thermal resistance films, as in the case of thick TE film samples, which brings a non-linear TR response to $(2\omega)^{0.5}$ and it is important to understand the thermal resistance films especially the films are multi-layers, like in our case.

The 150-nm-thick metal (Au) films with a size of 2 mm x 4 mm were formed on the surfaces of thin film samples. By applying a sinusoidal voltage with a frequency, f, of 200–10000 Hz between both ends of the Au films through the wires, electrical current flows resulting in the generation of Joule heating in the Au film. The periodic temperature change of the Au films was monitored by the TR signal in the center region of the Au films using a continuous wave He-Ne gas laser (k = 635 nm) with a beam diameter of 0.1 mm indicated in Figure S1.

First measurement

In order to evaluate the thermal conductivity of SiGe thin films, first we tried to exclude the effect of the substrate and to only get the SiGe properties, it is necessary to perform comparative evaluations with and without SiGe thin films, as shown in Figure S1. The samples of SiGe on Si_3N_4/SiO_2 multilayer on Si substrate and the substrate of Si_3N_4/SiO_2 multilayer on Si substrate were prepared. In this experiment, although we were able to measure the data, however, we were unable to accurately evaluate thermal conductivity. The obtained data of thermal conductivity were 11 to 13 $Wm^{-1}K^{-1}$, which was too high and was much higher than those of bulk SiGe. It is

thought that the thermal conductivity increased due to the contribution of the actual interfacial thermal resistance.

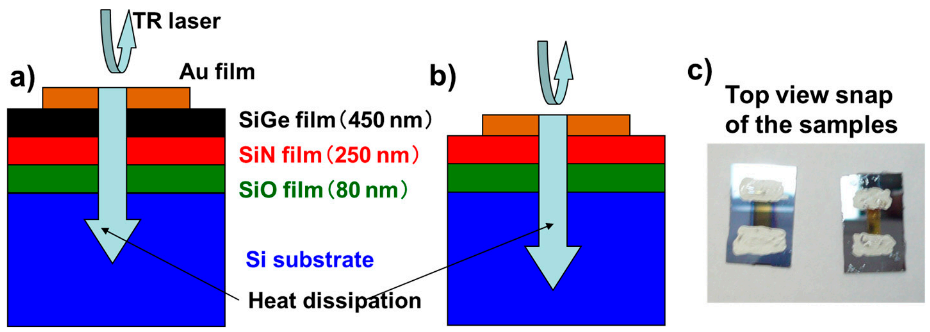

Figure S1. Measurement principle of the 2ω method for thin film on a substrate. A metal film is deposited on the thin film for Joule heating and TR measurement. (a) Schematic of Joule heat generated by the sinusoidal electrical current and its dissipation toward the substrate.

Second trial to obtain the reference data using a set of different SiGe film thickness samples

Assumed that the thermal resistance difference in the measurement results is due to the SiGe thin film thickness difference of 450 nm, then by preparing a thin film twice the original thickness, the interface thermal resistance can be excluded or reduced. In this case, it was assumed that the thermal conductivity of the thin film was constant and independent of film thickness. The advantage of this experiment is that the interfacial thermal resistance is completely canceled out.

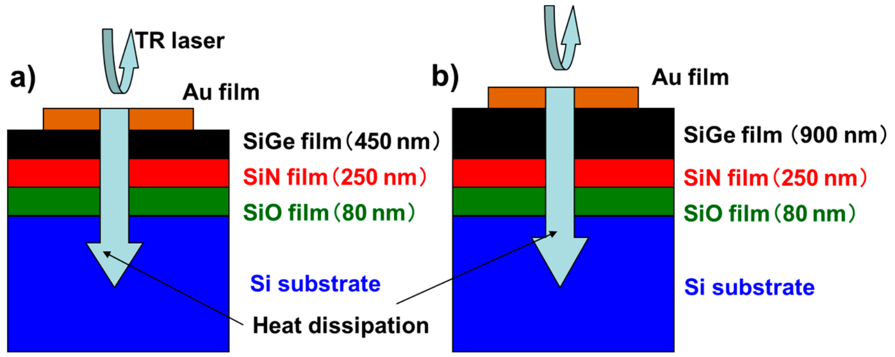

Figure S2. Modified measurement of the 2ω method for thin film on a substrate with SiGe of different thickness a) 450nm and b) 900nm.

From the comparison of two measured data a refence value of interfacial thermal resistance of $0.75 \pm 0.13 \times 10^{-7}$ m^2 K^{-1} is obtained which is used for the calibration of the thermal conductivity, the data of the first measurement are calculated again by other parameters of film thickness and heat capacity thermal conductivity of the layers [1] and calibrated as listed in **Table S1**.

Table S1. Thermal conductivity of 3 different boron-doping levels SiGe films on Si_3N_4/SiO_2 multilayer on Si substrate.

	Measured value	Calibrated by reference	
	Thermal resistance $m^2\ K\ W^{-1}$	Thermal resistance $m^2\ K\ W^{-1}$	Thermal conductivity $W\ m^{-1}\ K^{-1}$
B18	$(2.48 \pm 0.30) \times 10^{-7}$	$(1.73 \pm 0.22) \times 10^{-7}$	2.6 ± 0.4
B19	$(2.64 \pm 0.59) \times 10^{-7}$	$(1.89 \pm 0.36) \times 10^{-7}$	2.4 ± 0.6
B20	$(2.55 \pm 0.34) \times 10^{-7}$	$(1.80 \pm 0.24) \times 10^{-7}$	2.5 ± 0.4
B20-A	$(2.27 \pm 0.29) \times 10^{-7}$	$(1.52 \pm 0.21) \times 10^{-7}$	3.0 ± 0.5
reference	$(0.75 \pm 0.13) \times 10^{-7}$		

Reference

1. Okuhata, R., Watanabe, K., Ikeuchi, S. *et al.* Thermal Conductivity Measurement of Thermoelectric Thin Films by a Versatility-Enhanced 2ω Method. *J. Electron. Mater.* 46, 3089–3096 (2017). https://doi.org/10.1007/s11664-016-5170-5
